# Cocaine induced first-degree and high-grade second-degree atrioventricular block in a dog: a case report

**DOI:** 10.3389/fvets.2025.1622850

**Published:** 2025-08-18

**Authors:** Jake Johnson, Seth Bowden, Alyson H. Fitzgerald, Brittany D. Enders, Kursten V. Pierce

**Affiliations:** Department of Clinical Sciences, College of Veterinary Medicine, North Carolina State University, Raleigh, NC, United States

**Keywords:** bradyarrhythmia, arrhythmia, atrioventricular block, canine, drug, toxicosis

## Abstract

**Background:**

Common cocaine-induced arrhythmias include tachyarrhythmias such as sinus tachycardia, supraventricular tachycardia, and atrial fibrillation. Most studies evaluating cocaine toxicosis in canines have been performed in an experimental setting, using intravenous administration of the drug. Though helpful, these studies cannot be directly extrapolated in a clinical setting given the different routes of administration.

**Case details:**

A 2-year-old male-neutered Chihuahua presented for further management of acute onset of lethargy and a transient episode of unresponsiveness. Initial point of care ECG was consistent with an underlying sinus bradycardia with concurrent first-degree AV block and intermittent high-grade second-degree AV block. No murmur was noted on thoracic auscultation. Normal sinus rhythm returned after administration of atropine and epinephrine. Upon referral to a different facility for pacemaker implantation, sinus tachycardia was appreciated on point of care ECG without evidence of supraventricular or ventricular ectopy. On cardiac focused point of care ultrasound there was normal heart function and structure with no evidence of congenital heart defects. Both cardiac troponin and NT-proBNP were within normal limits. Urine toxicology was positive for cocaine, cocaine metabolites, norfentanyl and trace amounts of fentanyl. The patient was hospitalized overnight on telemetry, during which time infrequent ventricular premature complexes were the only abnormalities noted. The patient was discharged the following day.

**Conclusion:**

Cocaine-induced AV block in canines is an unusual presentation in a clinical setting, given the sympathetic stimulation this drug commonly causes. Emergency veterinary clinicians should be aware of this rare but important electrocardiographic abnormality following cocaine toxicosis.

## Introduction

Cocaine is a complex, illicit drug that acts as a central nervous system stimulant. Given its prevalence in North America, both human and veterinary medical facilities are familiar with treating patients for cocaine intoxication (1). As such, there are numerous studies in each respective field aimed at identifying and documenting the cardiovascular changes this drug may induce (1–6). Cocaine acts as a norepinephrine and dopamine reuptake inhibitor which produces sympathomimetic effects such as tachyarrhythmias (sinus tachycardia, supraventricular tachycardia, and atrial fibrillation). Common effects include life-threatening arrhythmias and conduction abnormalities, which may depend on the dose and route of intoxication (4, 5, 7, 8). In veterinary medicine, most studies analyzing canine cocaine intoxication have been performed in an experimental setting, using intravenous administration of the drug (1, 5, 8–10). Though helpful, these studies cannot be directly extrapolated in a clinical setting, given the different routes of administration. The present study highlights a novel clinical presentation and treatment of a dog that suffered from first-degree and high-grade second-degree atrioventricular (AV) block secondary to cocaine toxicosis.

## Case description and diagnostic assessment

A two-year-old, 5.5-kg, male-neutered Chihuahua was presented to an emergency clinic at 11:20 am for lethargy and unresponsiveness. A few minutes prior to arrival, the dog was found to be lethargic, unresponsive to audible stimuli, with his tongue protruding and poor visual tracking. The dog lives outdoors but does have access to the indoors and is current on core vaccines and preventatives. There are no known drug allergies or current medications.

On presentation, the animal was laterally recumbent with cyanotic mucous membranes, and initial vital signs included: bradycardia (32/min, reference interval [RI]: <60/min), eupnea (16/min, RI: 15-30/min), hypothermia (37.17°C, RI: 37.5–38.9°C [99.5–102.5°F], and normotension (oscillometric measurements of 124/92 mm Hg (MAP 103) and 101/83 mm Hg (MAP 89), respectively, RI: 110–160 mm Hg systolic and 60–90 mm Hg diastolic). No murmur or abnormal lung sounds were noted on thoracic auscultation.

**Figure 1:**
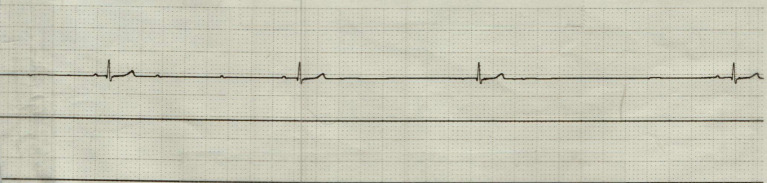
Point of care electrocardiogram (lead II; 50 mm/s) prior to administration of atropine and epinephrine. This represents bradycardia with concurrent first-degree AV block (PQ interval 140 ms) and intermittent high-grade second-degree AV block with a 3:1 conduction.

**Figure 2:**
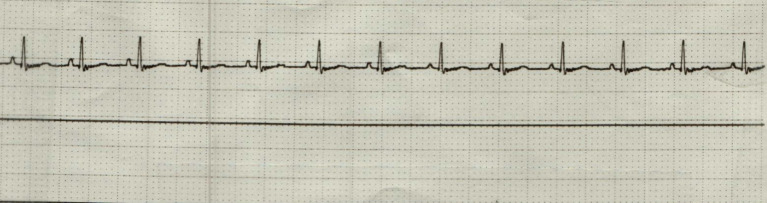
Point of care electrocardiogram (lead II; 50 mm/s) following administration of atropine and epinephrine. This represents a normal sinus rhythm with a heart rate of 144 bpm.

On complete blood count, there was equivocal hemoconcentration (hematocrit of 56%, RI: 37–55%) and a leukocyte count within normal range (8.79 × 10^9/L, RI: 6.0–17.0 × 10^9/L). Mild hyperglycemia (serum glucose of 164 mg/dL, RI: 60–110 mg/dL) was noted on chemistry analysis. On venous blood gas, acidemia (pH 7.159, RI: 7.32–7.44) with a respiratory acidosis (pCO2 53.8 mm Hg, RI: 26.0–45.0 mm HG) without metabolic compensation (HCO3 19 mmol/L, RI: 16.0–26.0 mmol/L); TCO2 21 mmol/L, RI: 16–26 mmol/L) was noted. Initial point of care ECG, recorded 31 min following presentation, was consistent with an underlying sinus bradycardia with concurrent first-degree AV block (PQ interval of 140 ms) and intermittent high-grade second-degree AV block with a 3:1 conduction interval (Figure 1). The patient was initially treated with a large, non-standard dose of atropine (0.2 mg/kg IV), which was followed up 9 min later, with a single bolus epinephrine (0.02 mg/kg IV). Point of care ECG recorded 1 h and 12 min following presentation was consistent with a normal sinus rhythm with a heart rate of 144 bpm (Figure 2). The patient was then referred to North Carolina State University Veterinary Teaching Hospital for further evaluation, monitoring, and consideration for pacemaker implantation if high-grade second-degree AV block persisted.

**Figure 3:**
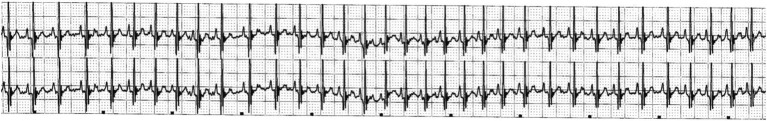
Point of care electrocardiogram (lead II; 25 mm/s) during presentation to the referral facility. This represents a sinus tachycardia (heart rate of 190 bpm).

Upon presentation to the transferring facility at 2:30 pm (2 h following discharge from the previous emergency center), the patient was bright, alert, and responsive. The only physical exam abnormalities noted were mydriatic pupils OU and tachycardia (190 bpm). Sinus tachycardia was confirmed on point of care ECG without evidence of supraventricular or ventricular ectopy (Figure 3). A cardiac focused point of care ultrasound performed by a cardiology resident SB, DVM (images reviewed by board-certified cardiologist KVP, DVM, DACVIM (Cardiology), FACVIM (Interventional Cardiology) revealed a structurally normal heart with normal valve leaflets, normal chambers sizes, and appropriate systolic function. Overall, normal heart function and structure with no evidence of congenital heart defects.

Repeat complete blood count and chemistry analysis were similar to those found at the referring emergency clinic. The patient’s previously noted acidemia and respiratory acidosis resolved based on repeat venous blood gas. The cardiac troponin and NT- proBNP, which were taken on arrival to the referral facility were within normal limits (<0.2 ng/mL (RI: <0.2 ng/mL) and 511 pmol/L (RI: <900 pmol/L), respectively). All parameters of a standard 4Dx test were negative. Urine was sampled shortly after presentation without complication via ultrasound-guided cystocentesis. Interestingly, the point of care urine drug screen using SAFElife™ T-Dip multi-drug urine test panel (immunoassay) was positive for cocaine. This sample (from initial urine collection) was sent to University of California, Davis for further toxicology screening via liquid chromatography with tandem mass spectrometry. The sample was positive for cocaine, cocaine metabolites (benzoylecgonine–positive and norcocaine–trace), fentanyl metabolite (norfentanyl) and trace amounts of fentanyl.

Further discussion with the owner revealed that the patient had 2 previous episodes of unknown drug toxicity as well as a history of dietary indiscretion and cryptorchidism, which was surgically fixated. The owners relayed that no items or medications were noted to be missing and that they did not have any controlled substances or illicit drugs in the house, though they had previously visited a friend and were uncertain if illicit substances were present in their house. There were several other prescription medications in the household in which ingestion could not be completely ruled out. However, the owners noted that these medications are secured and well-out of reach for potential ingestion. Of these medications, carvedilol would be the most likely to have led to the patient’s bradyarrhythmia. As a non-selective adrenergic receptor antagonist, carvedilol has effects against beta-1, beta-2, and alpha-1 receptors. Studies evaluating the pharmacokinetic effects of this medication in healthy dogs have appreciated dose-dependent decrease in heart rate following oral administration and dose-dependent increases in heart rate following intravenous administration. This increase is attributed to a decrease in systemic vascular resistance and activation of the baroreceptor reflex (11, 12). Given this data, as well as the unlikely possibility that the patient was exposed to this medication, the clinical presentation is thought to be unlikely to ingestion of this medication.

Following initial drug screen testing, the ASPCA Poison Control Center was called for consultation. General treatment recommendations included continued monitoring of body temperature, electrolytes, blood pH, and cardiovascular function. Specific drug interventions included: propranolol (0.02–0.06 mg/kg IV PRN) for management of tachycardia, diazepam (0.5–1.0 mg/kg in increments of 5–10 mg to effect) for tremors or seizure-like activity, and cyproheptadine (1.1 mg/kg PO or rectally, if vomiting) or acepromazine (0.1–1.0 mg/kg IV) for treatment of serotonin syndrome. Activated charcoal was recommended against while intralipid therapy was suggested if not improving with other treatments. Ultimately, it was suggested to continue until the patient remained asymptomatic without treatment for 6–8 h.

The patient was hospitalized overnight on telemetry to monitor heart rate and rhythm and maintenance crystalloid fluid therapy given overall adequate hydration levels on presentation (0.45% NaCl at 40 mL/kg/day with 0.05 mEq/kg/h of potassium chloride additive). During this time, infrequent ventricular premature complexes were the only abnormalities noted. The patient was discharged the following day with no medications. It was recommended that the owner continue to monitor for episodes of disorientation, overt lethargy, or any abnormal behaviors. Furthermore, the owners were advised to apply a basket muzzle to the dog during times when he could not be directly monitored in order to mitigate dietary indiscretion. The patient was subsequently lost to follow-up.

## Discussion

Given the patient’s history of dietary indiscretion, toxicology results, and clinical signs prior to referral, there is high clinical suspicion that the presenting bradyarrhythmia was due to a cocaine toxicosis. The two drugs detected on the urine toxicology screen were cocaine and fentanyl, as well as metabolites of each (13). Opioids at recommended doses are generally thought to have limited effects on cardiovascular stability, overdose of potent opioids may lead to profound bradycardia and respiratory depression, which can result in respiratory acidosis (13). Only a trace amount of fentanyl was detected on toxicology screening. A larger concentration of norfentanyl (a metabolite of fentanyl) was also detected. Fentanyl has an elimination half-life of about 3.5 h following buccal absorption; thus this patient would have had to ingest very large doses of fentanyl in order to be the primary toxicosis resulting in the bradyarrhythmia (14). Given the mentation and clinical presentation, this is deemed less likely. In one study, the elimination half-life of 4 mg/kg of orally absorbed cocaine was only 1.42 h (15). As such, this patient’s bradyarrhythmia is suspected to be caused primarily due to ingestion cocaine however it is important to note that cocaine is frequently adulterated with other drugs such as fentanyl thus it is not surprising that trace amounts of fentanyl were also detected.

To the author’s knowledge, high-grade second-degree AV block secondary to mucosal absorption of fentanyl has not been documented. In an experimental study of 24 dogs anesthetized with pentobarbital, intravenous doses of fentanyl at low (100 mcg/kg) and high (400 mcg/kg) doses prolonged the AH interval by 27 and 25%, respectively. The specific classes of AV block were not documented (16).

Cocaine is commonly considered a sympathomimetic, given its action as a neuronal norepinephrine and dopamine reuptake inhibitor. In humans, a wide range of arrhythmias have been documented secondary to acute cocaine intoxication. However, it most commonly results in tachyarrhythmias such as sinus tachycardia, re-entrant supraventricular tachycardia and less commonly, atrial fibrillation, ventricular tachycardia, and ventricular fibrillation (17). These findings are based on a conglomerate of small studies and case reports, since there is currently no individual study in human medicine that has systematically examined electrocardiographic findings in a large population of patients with acute cocaine toxicity. In veterinary medicine, there is a retrospective case series characterizing the clinical picture of 19 canines with presumptive cocaine toxicosis. In this cohort, sinus tachycardia was the only arrhythmia noted (*n* = 10/19) (1). Elevations in heart rate following acute cocaine toxicosis have also been wildly widely documented in other clinical and experimental reports (4, 5, 18). However, this response is not uniformly appreciated. Instead, there are various experimental reports (including mammals, humans and fish) that have documented bradyarrhythmias following acute cocaine toxicosis as well (5, 7, 18).

Bradyarrhythmias have been linked to cocaine’s inhibitory action on voltage gated sodium channels within the cardiac myocardium. Ultimately, this results in a pharmacologic potential similar to class I antiarrhythmics, thereby acting as a local anesthetic. This in turn causes prolongation of His bundle to ventricular duration and thus QRS duration (2, 5, 6, 19, 20). Additionally, cocaine inhibits potassium channels, particularly the rapidly activating delayed rectifier potassium channel, which leads to increased action potential duration and QT prolongation. As such, increases in intra-atrial and atrioventricular conduction time and atrial effective refractory period have all been documented (19).

Given the complex interaction of pharmacologic properties, cocaine-induced arrhythmias may be dose dependent in nature (4, 5, 8). A 2003 study documented the electrocardiographic effect that varying intravenous doses (low [1 mg/kg], medium [2 mg/kg], and high [5–7 mg/kg]) of cocaine had in 20 canines (5). The high dose group was the one where a significant electrocardiographic interval prolongation was appreciated compared to baseline. Interestingly, this was the only group where AV block was appreciated (type 1 [*n* = 2]; type II Mobitz type 1 [*n* = 1]; type II Mobitz type 2 [*n* = 1]; and type 3 [*n* = 1]).

Similarly, an experimental study of 8 anesthetized dogs with normal hearts showed that high-doses of cocaine slowed conduction in the atrium, AV node, His-Purkinje system, and ventricle, similar to class IB antiarrhythmic agents (lidocaine and mexiletine). However, lower doses, did not affect right intra-atrial conduction, QRS or QTc (21). Lastly, in a zebrafish study, comparable conclusions were made in which, acute low doses of cocaine increased heart rate while higher doses decreased it (7).

The canine in the present study had first-degree and high-grade second-degree AV conduction block. First-degree AV block occurs when conduction is delayed at the AV node, resulting in a prolonged PR interval beyond normal limits (>0.13 s in a dog) (22, 23). In contrast, second-degree AV block occurs when some sinus depolarizations pass through the AV junction to the ventricles while others do not. This type of block is further subdivided into type I, type II, and high-grade blocks. In type I (Mobitz type I or Wenckebach) block, the PR interval progressively lengthens before a blocked beat. The PR interval prior to the block may be normal or prolonged, as in first-degree AV block. In type II (Mobitz type II) block, conduction suddenly fails without changes in the PR interval, which may be normal or prolonged. Blocks with a 2:1 ratio or higher cannot be classified as type I or type II because there is no way to assess for progressive prolongation; these are referred to as high-grade blocks (23).

The presence of first-degree AV block cannot be detected clinically. As such, it is generally considered to have little clinical significance (22). In comparison, second-degree AV block is nearly always considered abnormal in a clinical setting, given a presumed increase in sympathetic stimulation. It may be caused by conduction system disease or physiologic and iatrogenic (e.g., digitalis and xylazine administration) processes that cause increased vagal tone, since the AV node is innervated with vagal fibers. Patients with type I or type II second-degree AV block typically show no clinical signs, so treatment is often unnecessary unless an underlying cause is identified and can be removed and needs to be addressed. Dogs with high- grade second-degree AV block may either show no clinical signs or present with symptoms similar to those of third-degree AV block, such as syncope and weakness (23).

Overall, AV block remains an uncommon result of cocaine toxicosis in canines. However, based on previous data, higher doses appear to be responsible for electrocardiographic interval prolongation and bradyarrhythmias (5, 7, 8). To explain this, it has been hypothesized that at higher doses, cocaine may have a direct toxic effect on the myocardium, thereby causing a decrease in heart rate. Reduced serum potassium levels may also be associated with higher doses of cocaine administration, which may result in more severe arrhythmias (5). However, in the presence of normal troponin and potassium on diagnostic testing results, both of these scenarios appear less likely in this case.

In the present study, it is unclear how much cocaine was ingested, nor the exact route of administration, though inhalation or ingestion appear likely. Based on plasma concentration bioequivalents used in a human study, the intravenous dose required to produce AV block was between 5 to 7 mg/kg (5). This equates to 96 mg or more of cocaine if intranasally ingested, given available data on plasma bioequivalents of intravenous administration in humans (23).

Human medical reviews of cocaine metabolism and plasma concentrations have reported that mucosal administration results in a slower onset of action, a later peak effect, and a longer duration of action as compared with the intravenous injection of cocaine (24, 25). In an experimental study involving 10 humans, participants received varying doses of cocaine via intravenous and intranasal routes. This showed a positive correlation between peak plasma concentration, physiological and subjective responses, and dose. Peak plasma concentrations following intravenous and intranasal administration of cocaine were seen at 8–12 min and 30–60 min, respectively.

Ultimately, heart rate returned to pre-drug levels in the IV group by 30 to 45 min. In contrast, the intranasal group reported the drug effect had disappeared within 60 to 90 min, which corresponded to return to pre-drug heart rate, without a concurrent decrease in cocaine plasma levels (24). Extrapolating from this data, this may indicate that the dog in the present study had come in contact with cocaine 60–90 min prior to when clinical signs were first appreciated.

An experimental study in humans geared toward understanding the elimination of cocaine and its metabolites in plasma, saliva, and urine following repeated oral administration found that peak cocaine concentration in urine occurs between 0.3–11.1 h after oral ingestion and the mean cocaine elimination half-life in urine is 19.0 h (range: 13.5–27.4). Interestingly, they noted the mean half-life for benzoylecgonine (cocaine metabolite) in urine was longer at 22.8 h (range: 15.0–34.5). As such, while variable, urine toxicology screenings for cocaine and its metabolites remain positive for around 3–5 days following exposure (28). However, this has been extended for up to 2 weeks in cases of chronic or repeated exposure. This is likely due to a slow release of cocaine that accumulates in fatty tissues during chronic use due to the lipophilic nature of the drug (25, 26).

The authors would like to acknowledge that the dose of atropine used to convert this patient’s rhythm was well above the typical recommended standard (0.04 mg/kg IV or SQ) dose (27). The elevated dose likely represents a deviation from standard practice and is recognized as a study limitation. Therefore, the response to standard atropine dosing remains unknown. As such, the authors are unable to comment on the response of this arrhythmia if the patient had been given the standard dose. This remains an inherent limitation of this study.

## Conclusion

The net cardiovascular effect of cocaine ingestion in canines remains complex. While cocaine typically acts as a sympathomimetic agent resulting in tachyarrhythmias, bradyarrhythmias have also been documented in experimental studies. To the author’s knowledge, the present study is the first clinical case report of cocaine toxicosis that resulted in first-degree and high-grade second-degree AV conduction block. This is likely due to the inhibitory action both voltage gated sodium and potassium channels have on the cardiac conduction system. As such, clinicians should consider cocaine exposure as a possible, though uncommon, cause of high-grade second-degree AV block in canines, specifically in cases where a higher ingestion dose is expected. This may be particularly important in patients that are being referred or considered for permanent pacemaker implantation for the AV block. Moving forward, more research into the long-term cardiovascular effects of cocaine toxicosis in canines is needed.

## Data Availability

The raw data supporting the conclusions of this article will be made available by the authors, without undue reservation.
